# Decreased GABA_B _receptor function in the cerebellum and brain stem of hypoxic neonatal rats: Role of glucose, oxygen and epinephrine resuscitation

**DOI:** 10.1186/1423-0127-18-31

**Published:** 2011-05-12

**Authors:** Thoppil R Anju, Sadanandan Jayanarayanan, Cheramadatikudiyil S Paulose

**Affiliations:** 1 Molecular Neurobiology and Cell Biology Unit, Centre for Neuroscience, Department of Biotechnology, Cochin University of Science and Technology, Cochin-682022 Kerala, India

**Keywords:** GABA_B_, neonatal hypoxia, cerebellum and brain stem

## Abstract

**Background-:**

Hypoxia during the first week of life can induce neuronal death in vulnerable brain regions usually associated with an impairment of cognitive function that can be detected later in life. The neurobiological changes mediated through neurotransmitters and other signaling molecules associated with neonatal hypoxia are an important aspect in establishing a proper neonatal care.

**Methods-:**

The present study evaluated total GABA, GABA_B _receptor alterations, gene expression changes in GABA_B _receptor and glutamate decarboxylase in the cerebellum and brain stem of hypoxic neonatal rats and the resuscitation groups with glucose, oxygen and epinephrine. Radiolabelled GABA and baclofen were used for receptor studies of GABA and GABA_B _receptors respectively and Real Time PCR analysis using specific probes for GABA_B _receptor and GAD mRNA was done for gene expression studies.

**Results-:**

The adaptive response of the body to hypoxic stress resulted in a reduction in total GABA and GABA_B _receptors along with decreased GABA_B _receptor and GAD gene expression in the cerebellum and brain stem. Hypoxic rats supplemented with glucose alone and with oxygen showed a reversal of the receptor alterations and changes in GAD. Resuscitation with oxygen alone and epinephrine was less effective in reversing the receptor alterations.

**Conclusions-:**

Being a source of immediate energy, glucose can reduce the ATP-depletion-induced changes in GABA and oxygenation, which helps in encountering hypoxia. The present study suggests that reduction in the GABA_B _receptors functional regulation during hypoxia plays an important role in central nervous system damage. Resuscitation with glucose alone and glucose and oxygen to hypoxic neonatal rats helps in protecting the brain from severe hypoxic damage.

## Background

Hypoxia is one of the most common reasons for neonatal morbidity and mortality, causing reduced oxygen supply to the vital organs [1] and injury to the developing brain [2-5]. The response of central nervous system to hypoxia is vital in revealing mechanisms that participate in coordinated behavior of respiratory and vasomotor activities [6,7].

The ventilatory response to acute hypoxia (hypoxic ventilatory response; HVR) in humans and some other mammalian species is biphasic [8,9]. The initial rise in ventilation (early phase of the HVR) is followed by a marked decline after several minutes to values above the prehypoxic level. This decline in ventilation has been termed "ventilatory roll-off" or "hypoxic ventilatory decline" (HVD). Several neurotransmitters and neuromodulators, such as γ-aminobutyric acid (GABA), [10-13] serotonin [14], adenosine, [15,16] and platelet-derived growth factor [17,18] play important roles in HVD. The alterations in neurotransmitter signaling in the respiratory control centers in brain stem and stressed breathing facilitating regions in cerebellar deep nuclei highly influence the ventilatory response of the body.

At synaptic transmission level, experimental hypoxia or hypoxia/ischemia increases the release of aminoacid neurotransmitters [19-23], causing an imbalance in normal activity of glutamatergic and GABAergic neurones, resulting in acute cell excitotoxicity. Endogenous GABA acting on GABA_A _or GABA_B _receptors modulates ventilation during room air breathing as well that the ventilatory response to acute and sustained hypoxia [24]. Rhythm generation in mature respiratory networks is influenced strongly by synaptic inhibition. Zhang et al, 2002 [24] reported that GABA_B_-receptor-mediated postsynaptic modulation plays an important role in the respiratory network from P0 on. GABA_B_-receptor-mediated presynaptic modulation develops with a longer postnatal latency, and becomes predominant within the first postnatal week [25].

GABA_B _receptors may contribute essentially to the modulation of respiratory rhythm in adult mammals and may be involved in the control of respiratory neuronal discharge [26]. GABA, which is metabolized in GABA shunts, is produced through α-decarboxylation of glutamic acid catalyzed by glutamate decarboxylase (GAD; EC 4.1.1.15) under the presence of cofactor pyridoxal 5'-phoshate. GAD, the rate limiting enzyme of GABA synthesis and a key protein in the GABA pathway, is used as a marker for GABAergic activity.

Thus, understanding the diagnosis, pathogenesis, resuscitation and treatment of those infants suffering hypoxic brain injury is paramount to reducing disability, improving survival and enhancing quality of life. Upon delivery, 5--10% of all newborns require some degree of resuscitation and assistance to begin breathing [27-29]. The aim of resuscitation is to prevent neonatal death and adverse long-term neurodevelopment sequelae associated with neonatal hypoxic event [30] and rapidly reverse fetal hypoxemia, and acidosis [31]. Debate regarding the optimal concentration of oxygen at initiation of resuscitation continues in the international community. The present study focused on understanding the alterations in GABA content, total GABA and GABA_B _receptors and GAD expression in the cerebellum and brain stem of hypoxic neonatal rats and the effects of various resuscitations on these alterations. The effectiveness of various resuscitation methods like administration of 100% oxygen and intravenous fluids like 10% glucose and 0.10 g/Kg body wt epinephrine alone and in combinations in the management of hypoxia was analyzed to understand the neuroprotective role of glucose supplementation. Understanding the molecular mechanisms involved in the regulation of neurotransmitter receptors will lead to better therapies for neonatal hypoxia-ischemia.

## Materials and methods

### Animals

Neonatal Wistar rats were purchased from Amrita Institute of Medical Sciences, Kochi. Neonatal rats of four days old were weighed and used for experiments. All groups of neonatal rat were maintained with their mothers under optimal conditions - 12 hour light and 12 hour dark periods and were fed standard food and water ad libitum. All animal care and procedures were taken in accordance with the institutional, National Institute of Health guidelines and CPCSEA guidelines.

### Induction of Acute Hypoxia in Neonatal Rats

Wistar neonatal rats of 4-days old (body weight, 6.06 ± 0.45 g) were used for the experiments and were grouped into seven as follows: (i) Control neonatal rats were given atmospheric air (20.9% oxygen) for 30 minutes (C); (ii) Hypoxia was induced by placing the neonatal rats in a hypoxic chamber provided with 2.6% oxygen for 30 minutes (Hx); (iii) Hypoxic neonatal rats were injected 10% dextrose (500 mg/Kg body wt) intra-peritoneally (i.p.) (Hx+G). (iv) Hypoxic neonatal rats were supplied with 100% oxygen for 30 minutes (Hx+O); (v) Hypoxic neonatal rats were injected 10% dextrose (500 mg/Kg body wt. i.p.) and treated with 100% oxygen for 30 minutes (Hx+G+O). (vi) Hypoxic neonatal rats were injected 10% dextrose (500 mg/Kg body wt), epinephrine (0.1 μg/Kg body wt. i.p.) and treated with 100% oxygen for 30 minutes (Hx+G+E+O) (vii) Hypoxic neonatal rats were injected with epinephrine (0.10 g/Kg body wt) i.p. (Hx + E). The experimental animals were maintained in the room temperature for one week.

### Tissue preparation

Control and experimental neonatal rats were sacrificed by decapitation. The cerebellum and brain stem were dissected out quickly over ice according to the procedure of Glowinski and Iversen, 1966 [32] and was stored at -80°C for various experiments.

### Quantification of GABA content Using [^3^H]Radioligands

GABA content in the cerebellum and brain stem of control and experimental rat groups was quantified by displacement method of Kurioka et al, 1981 [33] where the incubation mixture contained 30 nM [^3^H]GABA with and without GABA at a concentration range of 10^-8 ^M to 10^-4 ^M. The unknown concentrations were determined from the standard displacement curve using appropriate dilutions and calculated for μ moles/gm wt. of the tissue

### GABA Receptor Binding Assay

[^3^H] GABA binding to the GABA receptor was assayed in Triton X-100 treated synaptic membranes [33]. Crude synaptic membranes were prepared using sodium-free 10 mM tris buffer, pH 7.4. Each assay tube contained a protein concentration of 0.1 - 0.2 mg. In saturation binding experiments, 5 nM to 40 nM concentrations of [^3^H]GABA was incubated with and without excess of unlabelled GABA (100 μM) and in competition binding experiments the incubation mixture contained 30 nM of [^3^H] GABA with and without GABA at a concentration range of 10^-8^M to 10^-4^M were used.

### GABA_B _Receptor Binding Assay

[^3^H] baclofen binding to the GABA_B _receptor was assayed in Triton X-100 treated synaptic membranes [33]. Crude synaptic membranes were prepared using sodium-free 10 mM tris buffer, pH 7.4. Each assay tube contained a protein concentration of 0.1 - 0.2 mg. In saturation binding experiments, 5 nM to 40 nM concentrations of [^3^H]baclofen was incubated with and without excess of unlabelled baclofen (100 μM) were used.

Protein was measured by the method of Lowry et al, 1951 [34] using bovine serum albumin as standard.

### Linear regression analysis of the receptor binding data for Scatchard plots

The data was analysed according to Scatchard, 1949 [35]. The specific binding was determined by subtracting non-specific binding from the total. The binding parameters, maximal binding (B_max_) and equilibrium dissociation constant (K_d_), were derived by linear regression analysis by plotting the specific binding of the radioligand on X-axis and bound/free on Y-axis. The maximal binding is a measure of the total number of receptors present in the tissue and the equilibrium dissociation constant is the measure of the affinity of the receptors for the radioligand. The K_d _is inversely related to receptor affinity.

### Nonlinear regression analysis for displacement curve

Competitive binding data was analyzed using non-linear regression curve-fitting procedure (GraphPad PRISM™, San Diego, USA). The data of the competitive binding assays were represented graphically with the log of concentration of the competing drug on x-axis and percentage of the radioligand bound on the y-axis. The steepness of the binding curve can be quantified with a slope factor, often called a Hill slope. A one-site competitive binding curve that follows the law of mass action has a slope of 1.0 and a two site competitive binding curve has a slope less than 1.0. The concentration of competitor that competes for half the specific binding was defined as EC_50_, which is same as IC_50_. The affinity of the receptor for the competing drug is designated as K_i _and is defined as the concentration of the competing ligand that binds to half the binding sites at equilibrium in the absence of radioligand or other competitors.

### Gene expression studies in cerebellum and brain stem

RNA was isolated from the cerebellum and brain stem using Tri reagent. Total cDNA synthesis was performed using ABI PRISM cDNA Archive kit. Real-Time PCR assays were performed in 96-well plates in an ABI 7300 Real-Time PCR instrument (Applied Biosystems, Foster City, CA, USA). PCR analyses were conducted with gene-specific primers and fluorescently labeled Taq probe for GABA B (Rn 00578911) and GAD1 (Rn 00690304_g1) designed by Applied Biosystems. Endogenous control (β-actin) labeled with a reporter dye was used as internal control. All reagents were purchased from Applied Biosystems. The real-time data were analyzed with Sequence Detection Systems software version 1.7. All reactions were performed in duplicate.

The ΔΔCT method of relative quantification was used to determine the fold change in expression. This was done by first normalizing the resulting threshold cycle (CT) values of the target mRNAs to the CT values of the internal control β-actin in the same samples (ΔCT = CT _Target _- CT _β-actin_). It was further normalized with the control (ΔΔCT = ΔCT - CT _Control_). The fold change in expression was then obtained (2^-ΔΔCT^).

### Statistical analysis

The equality of all the groups was tested by the analysis of variance (ANOVA) technique for different values of p. Further the pair wise comparisons of all the experimental groups were studied using Students-Newman-Keuls test at different significance levels. The testing was performed using GraphPad Instat (Ver. 2.04a, San Diego, USA) computer program.

## Results

### GABA Content in the cerebellum and brain stem of control and experimental neonatal rats

The GABA content was decreased significantly (p < 0.001) in the cerebellum and brain stem of hypoxic neonatal rats compared to control. The decreased content was reversed to near normal in glucose supplemented groups - Hx + G and Hx + G + O (Table 1).

**Table 1:** GABA Content (μmoles/g wet wt.) in cerebellum and brain stem of Control and Experimental Groups of Neonatal Rats

Experimental groups	GABA Content (μmoles/g wet wt.)	
	
	Cerebellum	Brain stem
Control	6.45 ± 1.2	8.45 ± 1.8

Hx	2.02 ± 1.0^a^	4.06 ± 1.4^a^

Hx + G	6.25 ± 1.4 ^b^	9.85 ± 2.2 ^b^

Hx + G + O	6.60 ± 1.4 ^b^	8.66 ± 1.4 ^b^

Hx + O	3.55 ± 1.8 ^b^	6.01 ± 1.5 ^b^

Hx + E	3.05 ± 1.2 ^a^	4.55 ± 1.6 ^a^

Hx + G + E + O	3.12 ± 1.1 ^a^	5.02 ± 1.4 ^a^

Values are Mean ± S.E.M of 4-6 separate experiments. Each group consist 6-8 rats.

^a ^p < 0.001 when compared to Control

^b ^p < 0.001, ^c ^p < 0.01 when compared to hypoxic group

Hypoxic rats- Hx, Hypoxic rats glucose treated - Hx+G, Hypoxic rats oxygen treated - Hx+O, Hypoxic rats glucose and oxygen treated - Hx+G+O, Hypoxic rats epinephrine treated - Hx + E, Hypoxic rats glucose, epinephrine and oxygen treated - Hx+G+E+O

### Total GABA receptors in the cerebellum and brain stem of control and experimental neonatal rats

Receptor studies for total GABA showed a significant decrease in receptor number compared to control in the cerebellum and brain stem (*p *< 0.01, p < 0.001 respectively) of hypoxic neonatal rats. In glucose supplemented groups, Hx + G and Hx + G + O, the receptor number was reversed to near control (p < 0.001) in both the brain regions. Epinephrine supplemented groups, Hx + E and Hx + G + E + O, showed no significant reversal in the altered receptor number to control level. In Hx + O, the Bmax was significantly decreased (p < 0.001) compared to control (Table 2).

**Table 2:** Total GABA receptor binding parameters in the cerebellum and brain stem of control and experimental neonatal rats.

Experimental groups	Cerebellum		Brain stem	
	
	**B**_**max **_**(fmoles/mg protein)**	**K**_**d **_**(nM)**	**B**_**max **_**(fmoles/mg protein)**	**K**_**d **_**(nM)**
Control	71.50 ± 2.41	11.11 ± 0.95	153.36 ± 3.7	4.77 ± 0.44

Hx	50.01 ± 1.80 ^a^	14.82 ± 0.82 ^a^	116.68 ± 2.8 ^a^	3.77 ± 0.22 ^a^

Hx + G	62.18 ± 1.50 ^b^	9.85 ± 0.36 ^b^	173.36 ± 2.5 ^b^	6.78 ± 0.35 ^a, b^

Hx + G + O	66.33 ± 2.00 ^b^	12.54 ± 0.42	160.84 ± 3.4 ^b^	5.01 ± 0.26 ^a, b^

Hx + O	55.34 ± 2.50 ^a^	15.72 ± 0.54 ^a^	136.68 ± 2.3 ^a, b^	4.73 ± 0.29 ^b^

Hx + E	44.02 ± 3.20 ^a^	10.46 ± 0.10 ^b^	122.08 ± 2.6 ^a^	3.30 ± 0.14 ^a^

Hx + G + E + O	45.50 ± 2.50 ^a^	7.46 ± 0.11^a, b^	125.84 ± 4.5 ^a^	4.10 ± 0.22 ^b^

Values are Mean ± S.E.M of 4-6 separate experiments. Each group consist 6-8 neonatal rats.

^a ^p < 0.001 when compared with control

^b ^p < 0.001 when compared with hypoxic group.

Hypoxic rats- Hx, Hypoxic rats glucose treated - Hx+G, Hypoxic rats oxygen treated - Hx+O, Hypoxic rats glucose and oxygen treated - Hx+G+O, Hypoxic rats epinephrine treated - Hx + E, Hypoxic rats glucose, epinephrine and oxygen treated - Hx+G+E+O

### Non linear regression analysis of total GABA receptors in the cerebellum and brain stem

**Figure 1:**
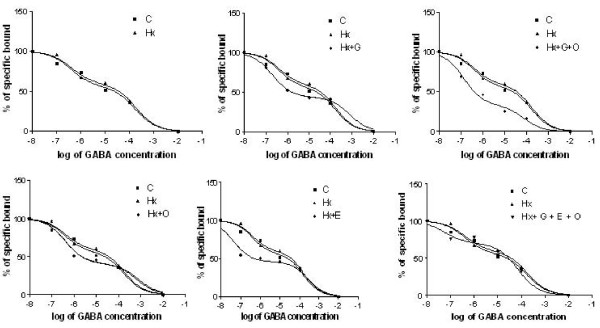
**Displacement of [^3^H] GABA against GABA in cerebellum of control and experimental neonatal rats**. Competition studies were carried out with 30 nM [^3^H] GABA in each tube with the unlabelled GABA concentrations varying from 10^-8 ^to10^-4 ^M. Values are representation of 4-6 separate experiments. Data from the curves as determined from nonlinear regression analysis using computer program PRISM fitted to a two-site model. The affinity for the first and second site for the competing drug is designated as Ki-1 (for high affinity) and Ki-2 (for low affinity). EC_50 _is the concentration of competitor that competes for half the specific binding. The equation built-in to the program is defined in terms of the log (EC_50_). If the concentrations of unlabelled compound are equally spaced on a log scale, the uncertainty of the log (EC_50_) will be symmetrical, but uncertainty of the EC50 will not be symmetrical

**Figure 2:**
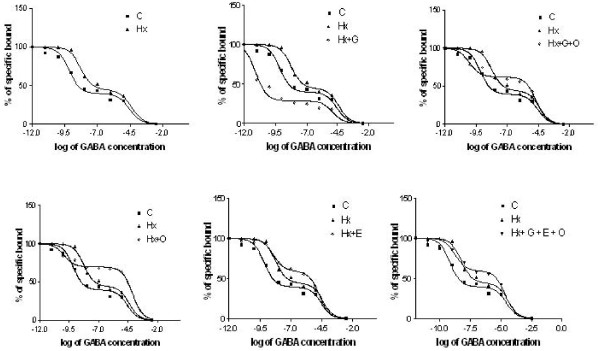
**Displacement of [^3^H] GABA against GABA in brain stem of control and experimental neonatal rats**. Competition studies were carried out with 30 nM [^3^H] baclofen in each tube with the unlabelled baclofen concentrations varying from 10^-12 ^to10^-4 ^M. Values are representation of 4-6 separate experiments. Data from the curves as determined from nonlinear regression analysis using computer program PRISM fitted to a two-site model. The affinity for the first and second site for the competing drug is designated as Ki-1 (for high affinity) and Ki-2 (for low affinity). EC_50 _is the concentration of competitor that competes for half the specific binding. The equation built-in to the program is defined in terms of the log (EC_50_). If the concentrations of unlabelled compound are equally spaced on a log scale, the uncertainty of the log (EC_50_) will be symmetrical, but uncertainty of the EC50 will not be symmetrical.

The binding data were confirmed by competition binding assay with [^3^H] GABA against different concentrations of GABA. GABA affinity in the cerebellum and brain stem of control and hypoxic neonatal rats fitted to a two site model with Hill slope value away from unity. GABA affinity of Hx + O, Hx + G, Hx + G + O, Hx + E and Hx + G + E + O also fitted to a two site model with Hill slope value away from unity. The Ki(H) increased in hypoxic neonatal rats along with an increase in the log (EC_50_)-1 indicating a shift in high affinity towards low affinity. Ki(L) also showed an increase in hypoxic neonatal rats with an increase in log (EC_50_)-2 denoting a shift in the low affinity site towards much lower affinity (Figure 1 &2).

### GABA_B _receptors in the cerebellum and brain stem of control and experimental neonatal rats

GABA_B _receptors was significantly decreased (p < 0.001) with a significant increase in its affinity (p < 0.001, p < 0.05) in the cerebellum and brain stem of hypoxic neonatal rats compared to control. Hx + G and Hx + G + O showed a significant reversal of B_max _(p < 0.001) and K_d _(p < 0.01) to near control in the cerebellum and a significant reversal of B_max _(p < 0.01, p < 0.001 respectively) to near control in the brain stem. In epinephrine and 100% oxygen supplemented groups, no reversal was observed (Table 3).

**Table 3:** GABA_B _receptor binding parameters in the cerebellum and brain stem of control and experimental neonatal rats.

Experimental groups	Cerebellum		Brain stem	
	
	**B**_**max **_**(fmoles/mg protein)**	**K**_**d **_**(nM)**	**B**_**max **_**(fmoles/mg protein)**	**K**_**d **_**(nM)**
Control	71.50 ± 2.41	11.11 ± 0.95	74.27 ± 1.20	13.31 ± 1.00

Hx	50.01 ± 1.80 ^a^	14.82 ± 0.82 ^a^	51.84 ± 1.50 ^a^	14.44 ± 0.99 ^b^

Hx + G	62.18 ± 1.50 ^b^	9.85 ± 0.36 ^b^	69.41 ± 1.40 ^b^	20.47 ± 0.99 ^a^

Hx + G + O	66.33 ± 2.00 ^b^	12.54 ± 0.42	70.47 ± 1.10 ^c^	26.10 ± 1.20 ^a^

Hx + O	55.34 ± 2.50 ^a^	15.72 ± 0.54 ^a^	49.10 ± 1.10 ^a^	16.36 ± 1.50 ^a^

Hx + E	44.02 ± 3.20 ^a^	10.46 ± 0.10 ^b^	43.59 ± 1.5 ^a^	14.53 ± 0.99 ^b^

Hx + G + E + O	45.50 ± 2.50 ^a^	7.46 ± 0.11^a, b^	53.95 ± 1.5 ^a^	13.90 ± 0.99 ^b^

Values are Mean ± S.E.M of 4-6 separate experiments. Each group consist 6-8 neonatal rats.

^a ^p < 0.001, ^b ^p < 0.05 when compared with control

^c ^p < 0.001 when compared with hypoxic group.

Hypoxic rats- Hx, Hypoxic rats glucose treated - Hx+G, Hypoxic rats oxygen treated - Hx+O, Hypoxic rats glucose and oxygen treated - Hx+G+O, Hypoxic rats epinephrine treated - Hx + E, Hypoxic rats glucose, epinephrine and oxygen treated - Hx+G+E+O

### Gene expression of GABA_B _receptor mRNA in the cerebellum and brain stem

**Figure 3:**
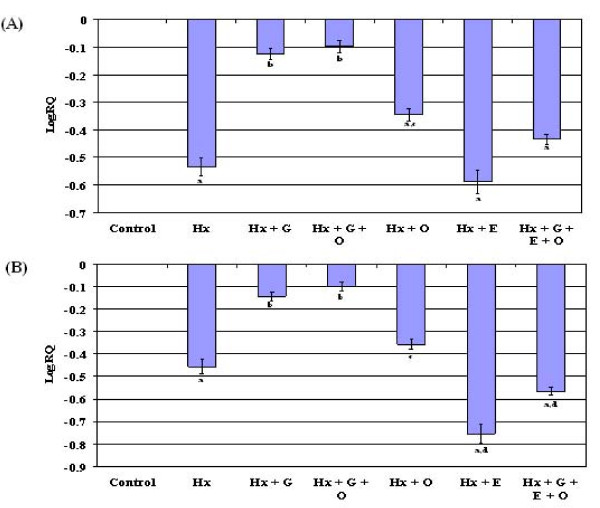
**Real time PCR amplification of GABA_B _receptor subunit in mRNA form the cerebellum (A) and brain stem (B) of control and experimental neonatal rats**. The ΔΔCT method of relative quantification was used to determine the fold change in expression. The relative ratios of mRNA levels were calculated using the ∆∆CT method normalized with β-actin. CT value as the internal control and Control CT value as the caliberator. PCR analyses were conducted in the cerebellum (A) and brain stem (B) with gene-specific primers and fluorescently labeled Taq probe GABA_B _(Rn 00578911)

GABA_B _receptor mRNA was significantly down regulated (p < 0.001) in the cerebellum and brain stem of hypoxic neonatal rats compared to control. In the cerebellum, Hx + G, Hx + G + O and Hx + O showed a significant reversal of GABA_B _receptor expression (p < 0.001, p < 0.001 and p < 0.05 respectively) to near control where as epinephrine supplemented groups, Hx + E and Hx + G + E + O, showed no significant reversal of altered expression. In the brain stem, glucose supplemented groups, Hx + G, Hx + G + O, showed a significant reversal of the gene expression (p < 0.001) to near control, whereas Hx + O, Hx + E and Hx + G + E + O showed a down regulated GABA_B _receptor expression (p < 0.01, p < 0.001, p < 0.001 respectively) with out a significant reversal to near control (Figure 3).

### Gene expression of GAD mRNA in the cerebellum and brain stem

**Figure 4:**
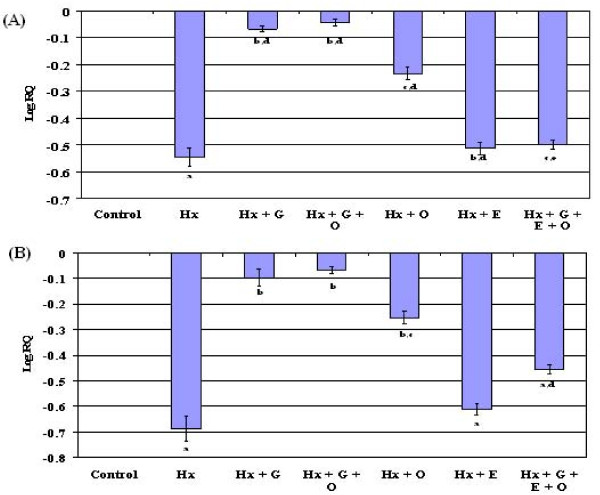
**Real time PCR amplification of GAD mRNA form the cerebellum (A) and brain stem (B) of control and experimental neonatal rats**. The ΔΔCT method of relative quantification was used to determine the fold change in expression. The relative ratios of mRNA levels were calculated using the ∆∆CT method normalized with β-actin. CT value as the internal control and Control CT value as the caliberator. PCR analyses were conducted in the cerebellum (A) and brain stem (B) with gene-specific primers and fluorescently labeled Taq probe GAD1 (Rn 00690304_g1).

The expression of glutamate decarboxylase in cerebellum and brain stem also showed a significant down regulation (p < 0.001) in the hypoxic group compared to control. The cerebellar and brain stem GAD expression was significantly reversed to near control in Hx + G, Hx + G + O and Hx + O whereas in Hx + E and Hx + G + E + O, there was no significant reversal to near control (Figure 4).

## Discussion

Hypoxia--ischemia (HI) occurring before or shortly after birth is a major cause of life-threatening injury and lifelong disability [36]. HI results in multi-organ failure and structural/functional damage especially devastating to the cardiovascular, renal, gastrointestinal and central nervous systems [37,38]. HI brain injury is very complex and has different neuropathological manifestations depending on the maturity of the newborn. Many of the structural changes that occur during the initial postnatal period in rodents are consistent with those seen during the late prenatal period in human brain development. Thus, exposure of rat to hypoxia on postnatal day 4 includes many of the neurodevelopmental events that may be affected by hypoxia in preterm human infants. In the present study, we investigated the functional regulation of GABA_B _receptors and GAD in hypoxic neonatal rats and the role of glucose, oxygen and epinephrine in altering the receptor status.

Numerous studies by different groups have confirmed that both inhibitory and excitatory amino acids are involved in the acute hypoxic ventilatory response [39-42]. Increases in GABA as a consequence of brain hypoxia can lead to depression of ventilation, which becomes more apparent in the absence of peripheral chemoreceptors. Blockade of GABA by biccuculine can significantly reduce this depressive effect of GABA on ventilation during hypoxia in chemodenervated animal or the newborn [43-45].

The present study reports a significant decrease in total GABA and GABA_B _receptor number with a down regulated receptor expression and glutamate decarboxylase expression in the cerebellum and brain stem regions of hypoxic neonatal rats. The decreased expression of GAD in turn results in the inhibition of GABA synthesizing pathway, which can be correlated to the decreased GABA receptors. The decreased GABA receptor is a response of the body to encounter hypoxic ventilatory decline. The reduction in GABA_B _receptor may help in overcoming the ventilatory decline during hypoxia but at the cost of severe central nervous system dysfunction. Louzoun-Kaplan et al, 2008 [46] reported that prenatal hypoxia at gestation day 17 in mice caused an immediate decrease in fetal cerebral cortex levels of glutamate decarboxylase. Decreased levels of key proteins in the GABA pathway in the cerebral cortex may lead to high susceptibility to seizures and epilepsy in newborns after prenatal or perinatal hypoxia. In the elevated plus maze, the agonist of GABA-B receptor was reported to improve consolidation of passive avoidance in rats undergoing hypoxia [47]. GABA_B _receptor-mediated activation of TASK-1 or a related channel provides a presynaptic autoregulatory feedback mechanism that modulates fast synaptic transmission in the rat carotid body [48]. The signaling cascade that triggers the altered transcription of GABA-B receptor and GAD under hypoxic stress can be related to the activation of apoptotic pathways by triggering Bax expression and deactivating CREB expression coupled with the activation of HIF. The accumulation of HIF-1α in ischemic or hypoxic tissues promote adaptive mechanisms for cell survival [49] and was found to be an important mediator of hypoxia-induced tolerance to ischemia [50]. Although HIF-1α is essential for adaptation to low oxygen levels, it has also been shown *in vitro *to mediate hypoxia-induced growth arrest and apoptosis [51]. The increased Hif 1 mRNA expression under hypoxia facilitates angiogenesis, vasodialation and erythropoiesis. But in severe hypoxic cases, HIF-1α is accumulated and leads to cell death by activating different target genes [52]. The role of HIF-1α in mediating pro death and pro survival responses, is dependent on the duration [53] and types of pathological stimuli [54] as well as the cell type that it induces [55].

We observed that glucose supplementation to hypoxic neonates alone and along with 100% oxygen showed a reversal in the altered GABA_B _receptor parameters and GAD expression in the cerebellum and brain stem. Glucose supplementation provides an instant source of energy to the brain cells thereby preventing ATP depletion mediated cell death. Hattori and Wasterlain, 2004 [56] observed a reduction in the blood glucose levels and substantially increased cerebral glucose utilization [57] as a result of hypoxic stress in experimental rats. Mónica Lemus et al, 2008 [58] reported that GABA_B _receptor agonist (baclofen) or antagonists (phaclofen and CGP55845A) locally injected into nucleus tractus solitarius modified arterial glucose levels and brain glucose retention.

The standard approach to resuscitation neonatal hypoxia is to use 100% O2. Further, resuscitation with 100% is recommended as a beneficial short-term therapy that is generally thought to be non-toxic [31,59]. Although the use of 100% O2 appears intuitive to maximize the gradient required to drive O2 into hypoxic cells [30], a building body of evidence derived from animal models, has demonstrated that although resuscitation with 100% O2 improves restoration of cerebral and cortical perfusion, it may occur at the price of greater biochemical oxidative stress [31]. Resuscitation with 100% O_2 _significantly increased glutamate and glycine in the dorsal cortex contralateral to the ligated common carotid artery, compared to piglets resuscitated with 21% O_2_. These data suggest that persistent changes in neurochemistry occur 4 days after hypoxic ischemia and further studies are warranted to elucidate the consequences of this on neonatal brain development [60]. We observed that 100% oxygen resuscitation for neonatal hypoxia is not as effective as the combination of glucose and oxygen or administration of glucose alone. In cerebellum and brain stem of 100% oxygen resuscitated groups, GABA_B _receptors showed a significant decrease compared to control. One hundred percentage of oxygen generated abnormally high levels of reactive oxygen species (ROS) which causes dysfunction of defensive antioxidant system of cells by altering enzyme activity [61,62] and act as a factor for neurodegeneration [63]. Hypoxemic piglets resuscitated with 100% O_2 _also showed increased cerebral injury, cortical damage and early neurologic disorders [64-66]. Previous studies on acetylcholinesterase [67], GABA_A _and serotonin receptors [68] reported the neuroprotective role of glucose and combination of glucose and oxygen resuscitation and the damaging effects of oxygen supplementation alone. The reduction in GABA_B _receptor number in the cerebellar and brain stem regions during oxygen supplementation is suggested to be due to tissue damage caused by the formation of free radicals or reactive oxygen species and the changes in amino acids resulting in neuronal cell death. During oxygen resuscitation, the accumulation of ROS activates the over stimulation of HIF 1 alpha which can in turn results in the activation of apoptotic pathways by altering the expression of transcription factors like CREB and NF-Kappa-B.

Epinephrine is routinely used in the resuscitation for persistent severe neonatal hypoxia. The present study points out the adverse effects of epinephrine supplementation, alone and even in combination with glucose and oxygen, by studying the changes in GABA_B _receptor, expression of GABA_B _receptor and GAD in the brain stem and cerebellum. The GABA_B _receptor was significantly decreased in epinephrine treated groups. A reflex action of epinephrine firing occurs during hypoxia. Supplementation of epinephrine to already excited system results in its hyper activity and it affects the balance of various neurotransmitters like dopamine [69] and glutamate. Epinephrine induces a hypoxia-neovascularization cascade and plays a primary role in vascular proliferation within soft tissues [70]. It is reported that repetitive hypoxic stress induced by labour is a powerful stimulus for catecholamine release in fetus and is accompanied by typical alterations of fetal heart rate. The high influx of this excitatory neurotransmitter affects the balance of other neurotransmitters thereby disrupting the cascade of signal transduction.

There has been much interest in the acute neurological changes associated with neonatal hypoxia, along with the mechanisms of subsequent central nervous system dysfunction in the adult [71-74]. Hypoxia during the first week of life can induce neuronal death in vulnerable brain regions usually associated with an impairment of cognitive function that can be detected later in life [75]. Postnatal hypoxia resulting from lung immaturity and respiratory disturbances in infants is an important pathophysiological mechanism underlying the devastating neurological complications. This points the importance of a proper resuscitation program to overcome neonatal hypoxia for a better intellect in the later stages of life.

## Conclusions

Our studies point out the neuroprotective role of glucose in the management of neonatal hypoxic stress. The down regulated GABA_B _receptor in cerebellum and brain stem led to hypoxia induced ventilatory decline and activation of apoptotic pathways. These receptor alterations are reversed back to near control by the timely resuscitation with glucose, alone and in combination with oxygen. The deleterious effect of oxygen alone and epinephrine resuscitation in neuronal response through alterations in neurotransmitters was also observed. Thus it is suggested that glucose administration immediately after hypoxia with oxygenated air as a resuscitation programme will be of tremendous advantage especially in neonatal care. Deeper understanding of mechanisms, through which hypoxia regulates the neurotransmitters, could point towards the development of new therapeutic approaches to reduce or suppress the pathological effects of hypoxia.

## Competing interests

The authors declare that they have no competing interests.

## Authors' contributions

TRA carried out the receptor assays, gene expression and drafted the manuscript. SJ participated participated in the design of the study and performed the statistical analysis. CSP conceived of the study and participated in its design and coordination. All authors read and approved the final manuscript.
